# Supplementary material for: MYH10 activation rescues contractile defects in arrhythmogenic cardiomyopathy (ACM)
Source: Nat Commun. 2023 Oct 13;14:6461. doi: 10.1038/s41467-023-41981-5 (PMC10575922; doi:10.1038/s41467-023-41981-5)
Supplement: Supplementary file 1 — Supplementary Information [file 41467_2023_41981_MOESM1_ESM.pdf]

Supplementary Table 1

Mean ± SD	**RV-EDV [μL]**	**RV-ESV [μL]**	**RV-EF [%]**	**LV-EDV [μL]**	**LV-ESV [μL]**	**LV-EF [%]**
**Sham**	63.17 ± 6.6	20.00 ± 6.1	71.17 ± 3.5	66.33 ± 5.6	21.33 ± 7.4	68.50 ± 8.8
**PKP2**	66.00 ± 15.8	20.67 ± 2.4	69.50 ± 3.8	67.00 ± 12.4	22.33 ± 5.8	67.50 ± 3.7
**PKP2-p.R735***	61.67 ± 7.6	28.67 ± 4.6	57.83 ± 5.3	64.33 ± 8.2	25.00 ± 9.3	65.00 ± 7.2

Supplemental Table 1 PKP2 C-terminal deletion modifies systolic function
Cardiac MRI data from non-transduced control mice and transduced with AAV-PKP2, AAV-PKP2-p.R735*. EDV: End Diastolic Volume; ESV: End Systolic Volume; RV: Right ventricle; LV: Left ventricle; EF: ejection fraction. SD: standard deviation; μL: microliters; %: percentage. RV: right ventricle; LV: left ventricle; EDV: end diastolic volume; ESV: end systolic volume; EF: ejection fraction. Source data are provided as a source data file

Supplementary Table 2

Mean ± SD	**RV-EDV [μL]**	**RV-ESV [μL]**	**RV-EF [%]**	**LV-EDV [μL]**	**LV-ESV [μL]**	**LV-EF [%]**
**Sham**	66.70 ± 11.75	20.0 ± 6.12	70.60 ± 3.03	68.70 ± 7.26	20.70 ± 6.48	70.20 ± 8.31
**EGFP-PKP2**	66.10 ± 12.78	21.40 ± 4.81	68.80 ± 5.39	68.60 ± 10.19	22.40 ± 5.64	67.20 ± 4.54
**EGFP-PKP2-p.R735***	67.80 ± 10.66	29.00 ± 5.35	58.00 ± 6.02	69.00 ± 8.91	26.30 ± 8.98	63.20 ± 7.73
**PKP2-p.R735*-EGFP**	66.13 ± 7.10	24.50 ± 4.24	64.13 ± 5.64	67.88 ± 11.10	21.75 ± 6.18	66.50 ± 6.53

Supplemental Table 2 PKP2 N-terminal misfolding recovery ameliorates cardiac dysfunction
Cardiac MRI data from non-transduced control mice and transduced with AAV-EGFP-PKP2, AAV-EGFP-PKP2-p.R735* or AAV-PKP2-p.R735*-EGFP. Panel shows quantification of. EDV: End Diastolic Volume; ESV: End Systolic Volume; RV: Right ventricle; LV: Left ventricle; EF: ejection fraction. Data are presented as mean ± sd; (n=8, n=10 and n=10 animals respectively, from 3 independent experiments). SD: standard deviation; μL: microliters; %: percentage; RV: right ventricle; LV: left ventricle; EDV: end diastolic volume; ESV: end systolic volume; EF: ejection fraction. Source data are provided as a source data file.

Supplementary Figure 1

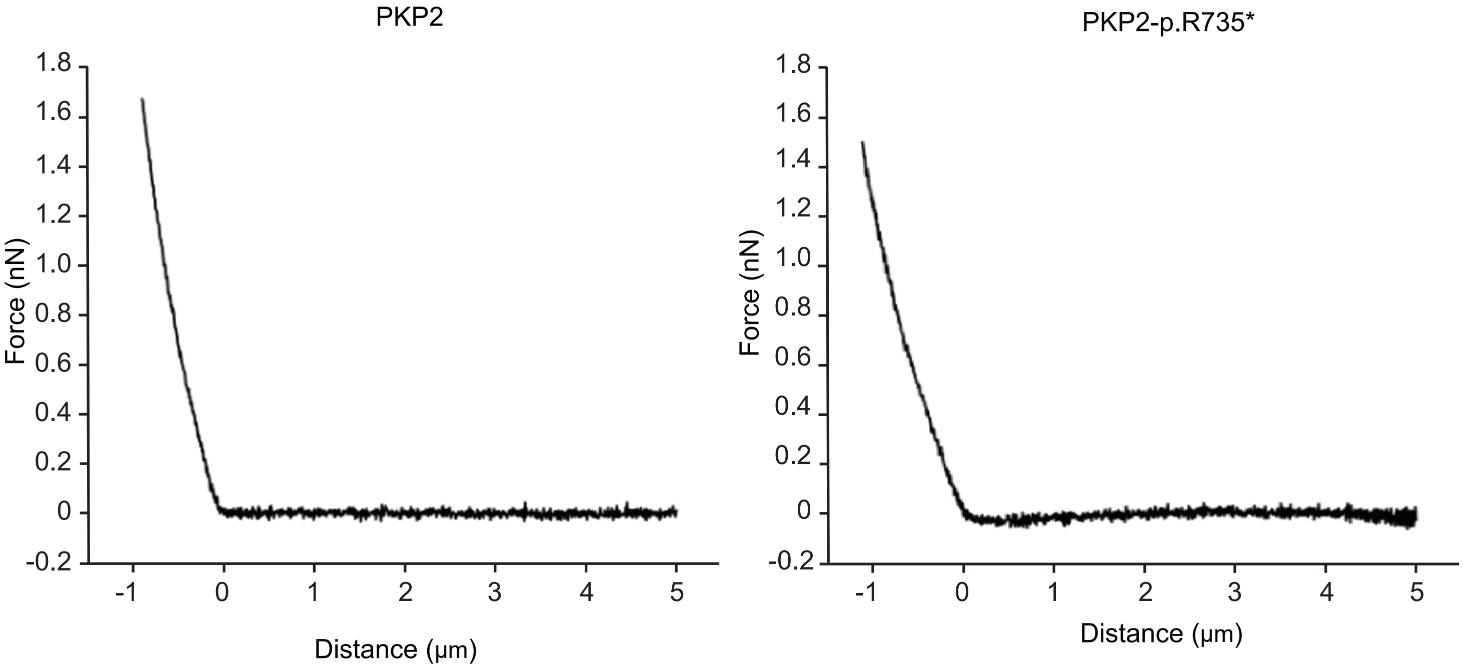

Supplemental Fig. 1 PKP2 mutant modifies cellular stiffnes
Illustrative fitting force-distance curves from force maps of HL-1 cells expressing PKP2 or the R735* mutant generated from AFM measurements, showing the maximum indentation of the cell surface used to calculate Young's modulus. nN: nanoNewtons; μm: micrometers. Source data are provided as a source data file.

Supplementary Figure 2

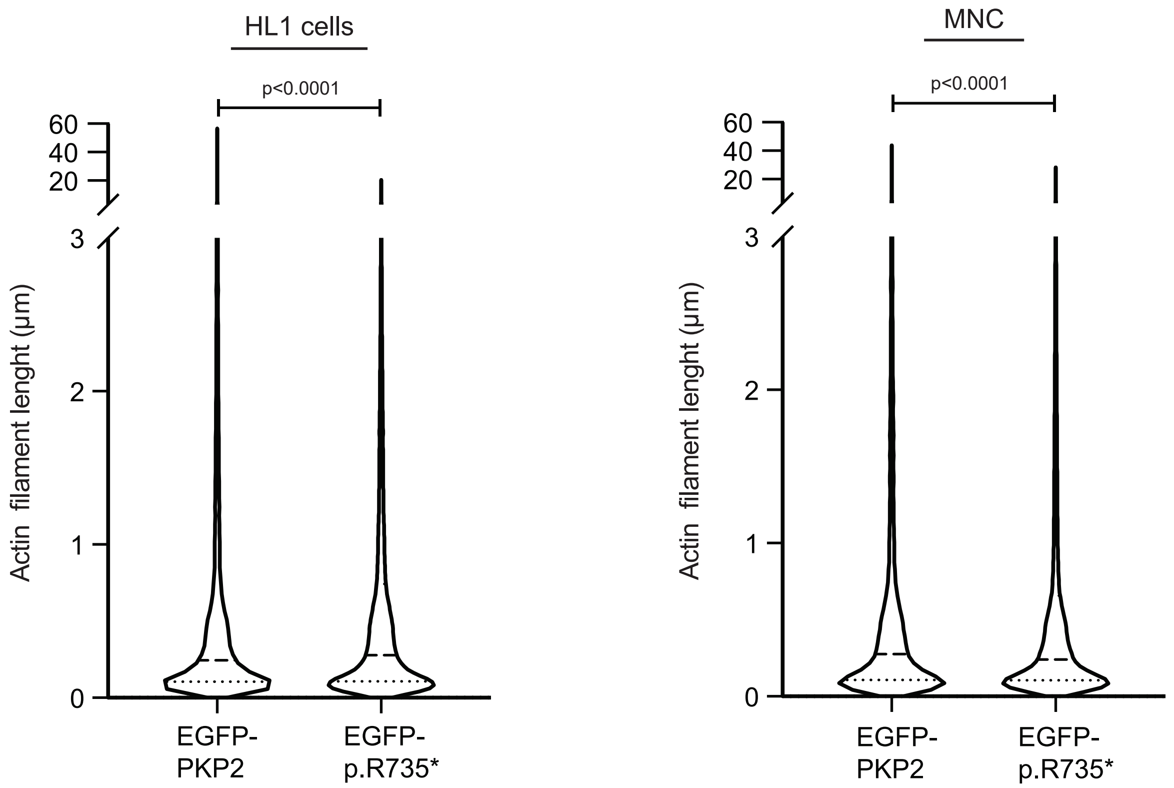

Supplemental Fig. 2 PKP2 mutant reduces actin filament length

Violin plots showing quantification from confocal images of actin filament length in HL-1 cells and mouse neonatal cardiomyocytes (MNC). Statistical significance was determined by one-way ANOVA with Tukey's multiple comparison post-test with $p<0.05$ considered statistically significant. Data are presented as mean ± sem. µm: micrometers. Source data are provided as a source data file.

Supplementary Figure 3

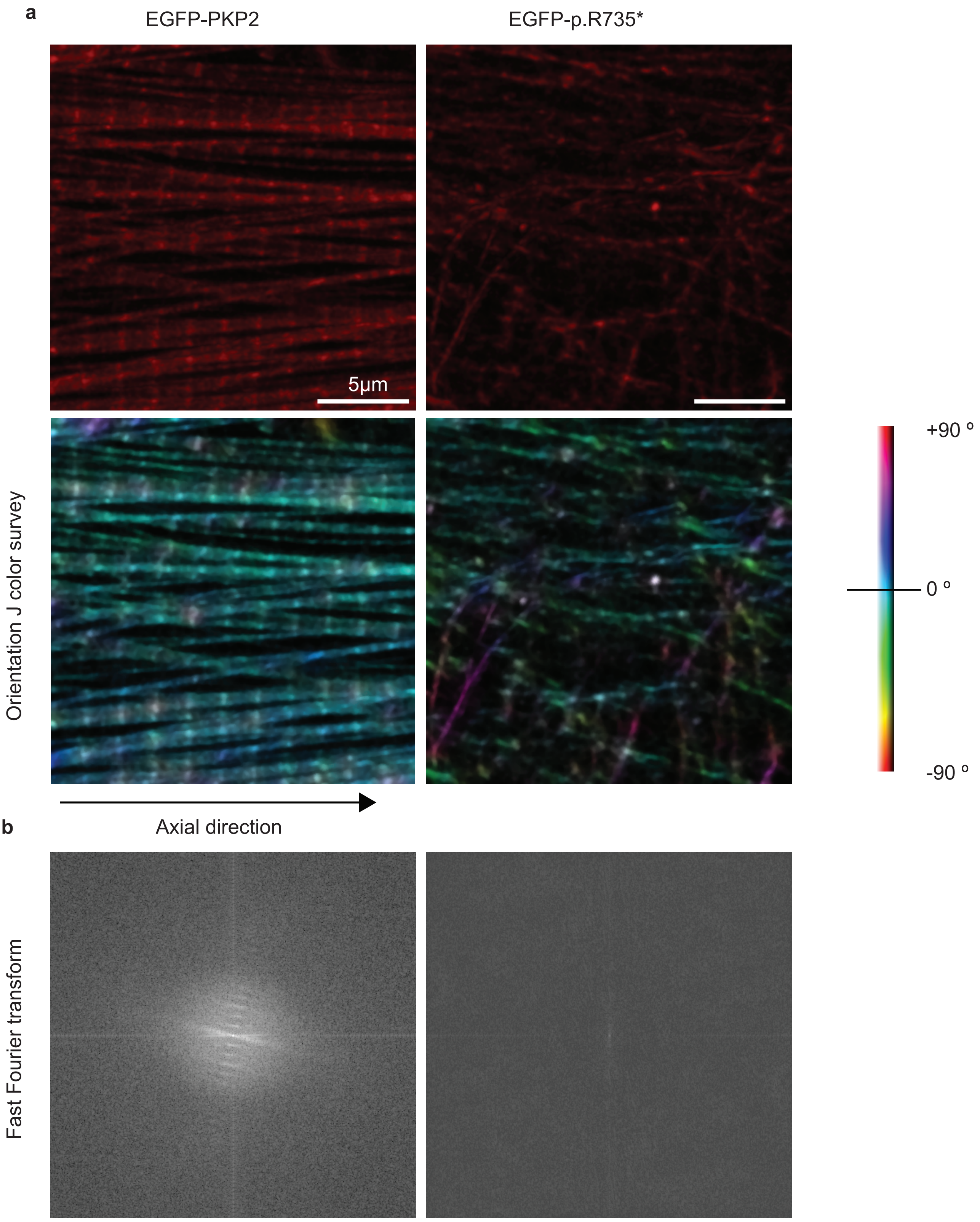

Supplemental Fig. 3 PKP2-p.R735* alters direction of the actin fibers in space
a, Confocal representative image of F-actin of MNC samples transfect with EGFP-PKP2 or EGFP-PKP2-p.R735*. Hue-saturation-brightness (HSB) color-coded map from OrientationJ plugin of ImageJ which shows the orientation of the actin fibers in space relative to the axial direction. b, 2D fast Fourier transform (FFT) spectrum of image in a with its gray level profile from TTorg plugin of ImageJ. Scale bar 5 μm. Source data are provided as a source data file.

Supplementary Figure 4

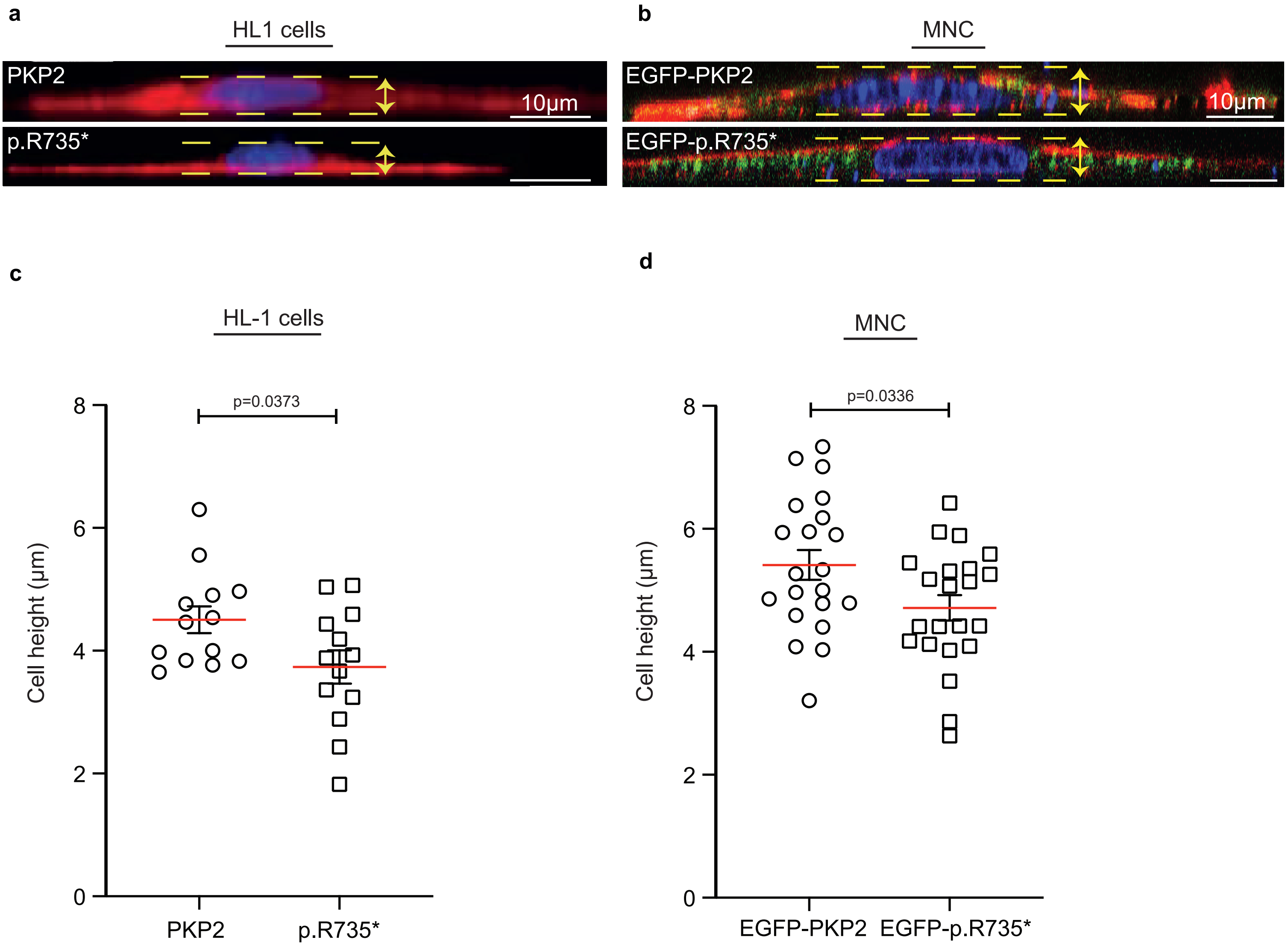

Supplemental Fig. 4 Abnormal height of PKP2-p.R735* cells
a, Side views of HL-1 cells or (b) mouse neonatal cardiomyocytes (MNC) expressing PKP2 or the p.R735* mutant (green) generated by transverse maximal projection of F-actin (red) and nuclei (blue) staining. Cell height measured from confocal sections taken from the base to the top of phalloidin-stained control cells or cells expressing PKP2 or p.R735*. c, d, Charts plotting maximum cell height corresponding to separation of the yellow lines in the images represented as an example from HL-1 cells or MNC. Data are presented as mean ± sem; n=13 HL-1 cells, n=22 MNC cells, repeated twice. Statistical significance was determined by unpaired t test with Welch´s corrections (two-sided) with $p<0.05$ considered statistically significant. Data are presented as mean ± sem. μm: micrometers. Source data are provided as a source data file.

Supplementary Figure 5

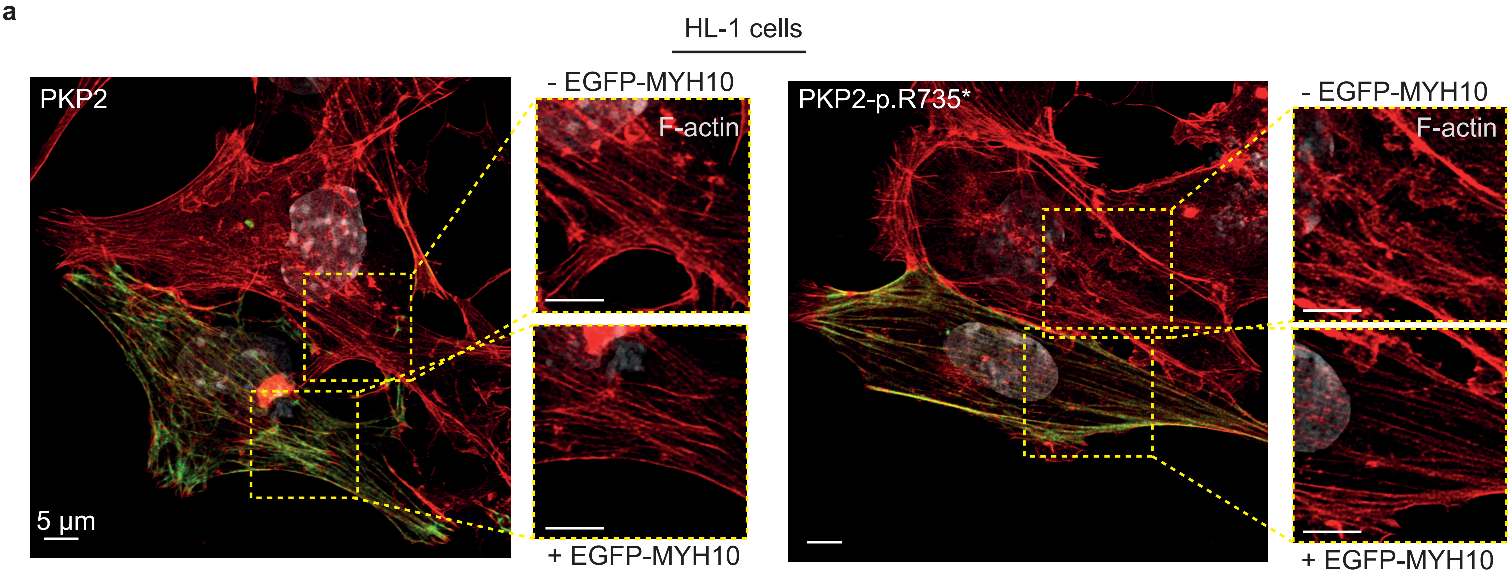

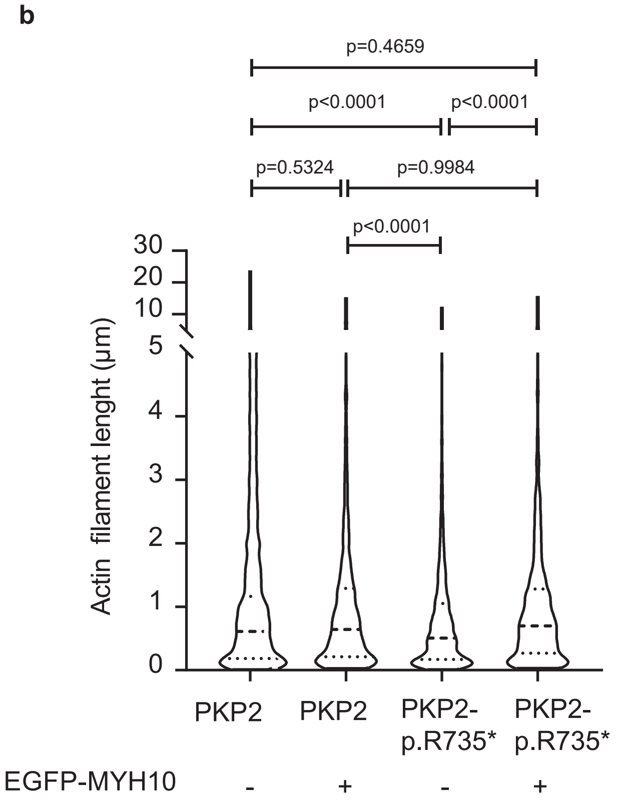

Supplemental Fig. 5 MYH10 significantly improved actin network in cells expressing PKP2-p.R735* mutant a, Representative images of actin filaments (red) in HL-1 stable cells expressing PKP2 or PKP2-p.R735* together with functional EGFP-MYH10 (green). Right panels show a magnification of actin filaments in cells with or without EGFP-MYH10. b, Violin plot shows quantification from confocal images of actin filament length in HL-1 (n= 3 cells from 3 independent experiments). Statistical significance was determined by one-way ANOVA with Tukey's multiple comparison post-test with p<0.05 considered statistically significant. Data are presented as mean ± sem. Scale bars, 5 µm. m: micrometers. Source data are provided as a source data file.

Supplementary Figure 6

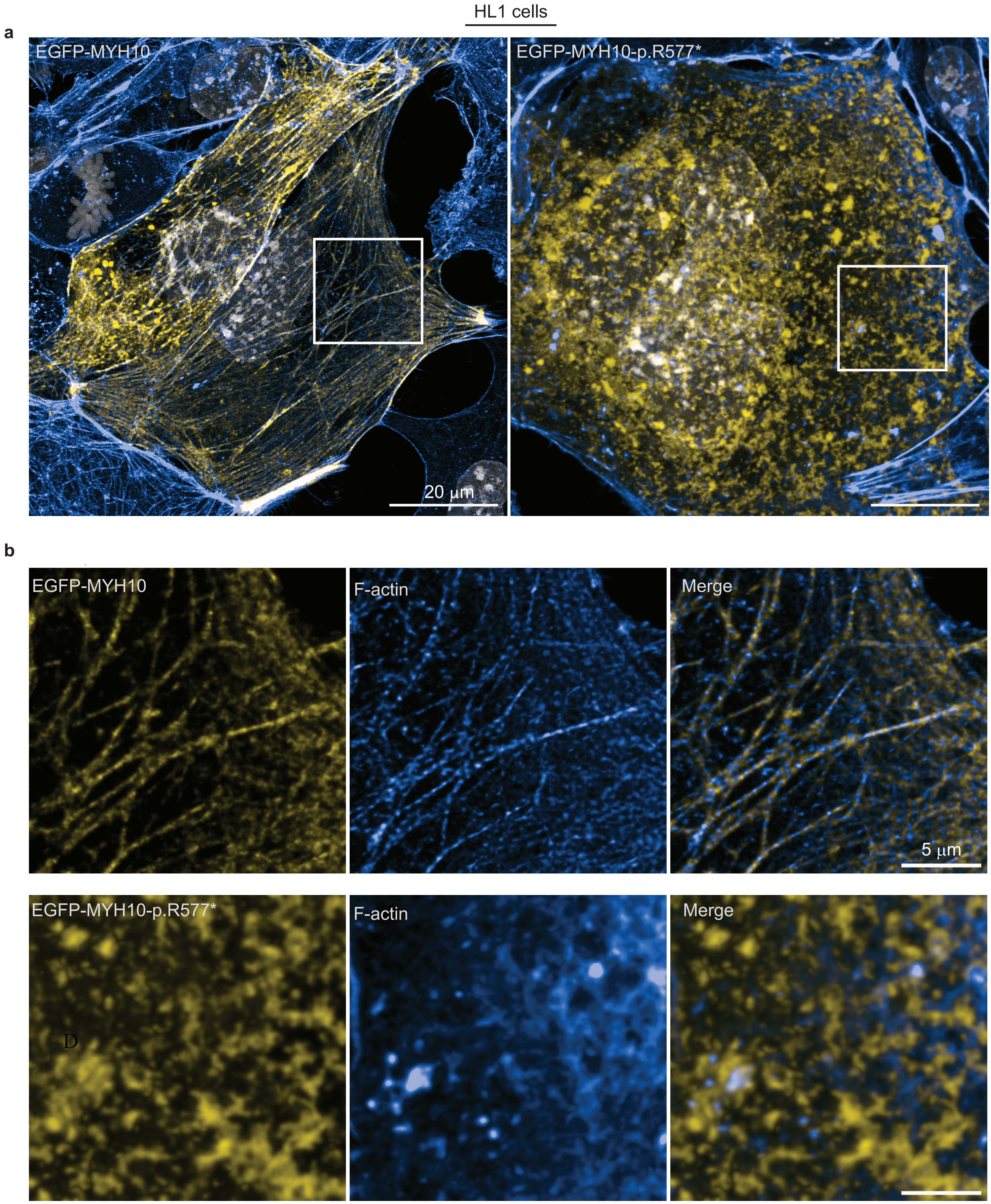

Supplemental Fig. 6 MYH10 forms distribution along the F-actin fibers
a, Representative confocal images showing phalloidin-staining of the F-actin network (blue) in HL-1 cells expressing wildtype EGFP-MYH10 or EGFP-MYH10-p.R577* mutant (yellow). Nuclei are show in grey. b, A magnification of selected area (square) showing wildtype or mutant (R577*) Myh10, F-actin and merged signals. Scale bars, 20 µm and 5 µm. µm: micrometers. Source data are provided as a source data file.

Supplementary Figure 7

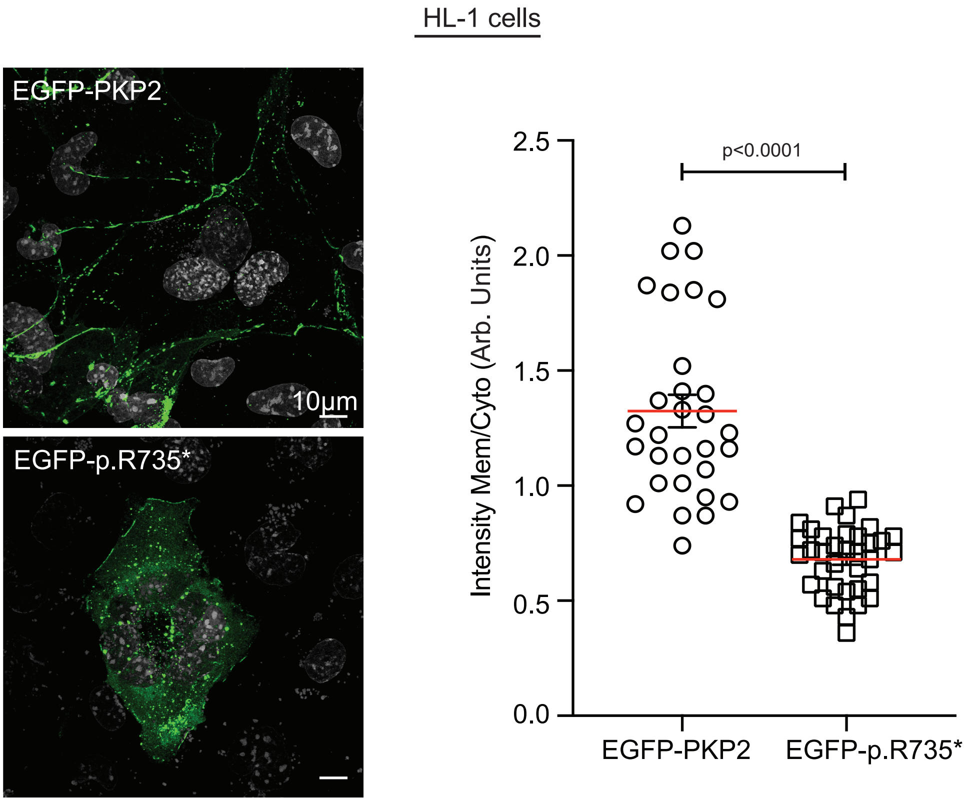

Supplemental Fig. 7 PKP2 C-terminal deletion mutant shows an altered subcellular localization

Representative images of showing the distribution in HL-1 cells of EGFP-PKP2 or EGFP-PKP2-p.R735* mutant. Scale bars, 10µm. b, Dot-plot showing the distribution of PKP2 versions as ratio intensity of membrane-cytoplasm. Data are presented as mean ± sd; n=30 EGFP-PKP2, and n=35 EGFP-PKP2-p.R735* mutant cells respectively, from 3 independent experiments. Statistical significance was determined by unpaired t test with Welch´s corrections (two-sided) with $p<0.05$ considered statistically significant. Arb. Units: arbitrary units; m: micrometers. Source data are provided as a source data file.

Supplementary Figure 8

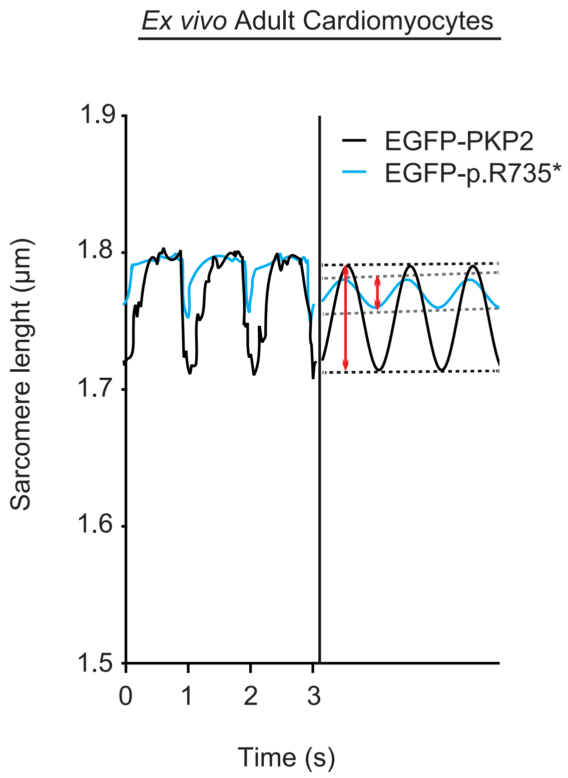

Supplemental Fig. 8 Cardiomyocytes encoding PKP2-p.R735* mutant presents sarcomeric contractile dysfunction
Graph showing pacing heartbeat (left) with its schematic representation (right) of contraction in adult isolated cardiomyocytes from animals transduced with AAV-EGFP-PKP2 or AAV-EGFP-PKP2-p.R735*. µm: micrometer; s: seconds. Source data are provided as a source data file.

Supplementary Figure 9

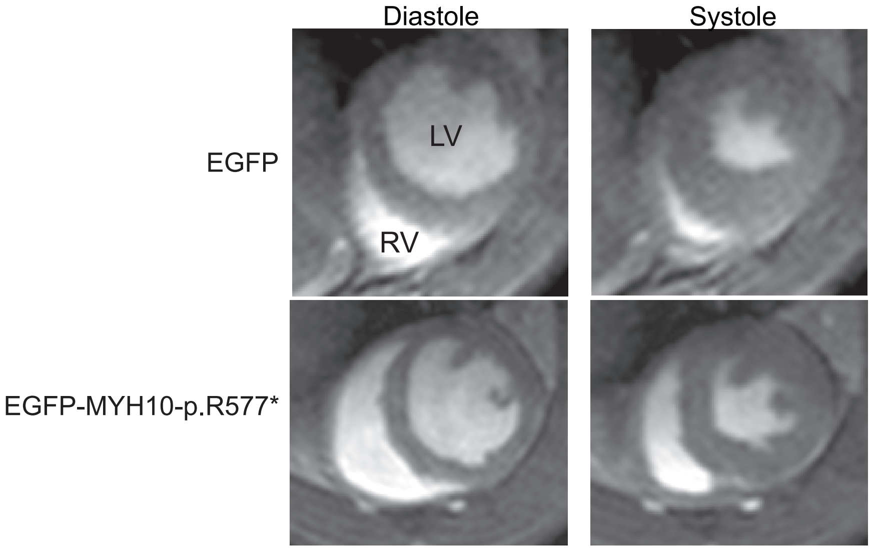

Mean ± SD	RV-EDV [µL]	RV-ESV [µL]	RV-EF [%]	LV-EDV [µL]	LV-ESV [µL]	LV-EF [%]
**Sham**	33.20 ± 5.16	11.70 ± 3.34	64.90 ± 5.36	56.40 ± 6.35	16.60 ± 4.20	70.70 ± 4.81
**EGFP**	30.60 ± 8.56	9.80 ± 3.03	68.20 ± 3.56	53.00 ± 13.53	13.80 ± 4.82	74.40 ± 4.98
**EGFP-MYH10-p.R577***	34.20 ± 9.43	17.30 ± 5.93	49.80 ± 5.35	61.20 ± 9.73	19.80 ± 6.44	68.10 ± 6.94

Supplemental Fig. 9 Mice encoding MYH10-R577* mutant present systolic dysfunction
Representative short-axis cardiac MRI images taken at the end of diastole and systole of indicated mice. Lower panel summarizes the cardiac function data determined by MRI in hearts of non-transduced (sham) and AAV-EGFP or AAV-EGFP-MYH10-p.R577* transduced mice. EDV: End Diastolic Volume; ESV: End Systolic Volume; RV: Right ventricle; LV: Left ventricle; EF: ejection fraction. Data are presented as mean ± sd; n=10 mice for all the groups except in sham n=5. LV: left ventricle; RV: right ventricle; SD: standard deviation; µL: microliters; %: percentage; RV: right ventricle; LV: left ventricle; EDV: end diastolic volume; ESV: end systolic volume; EF: ejection fraction. Source data are provided as a source data file.

Supplementary Figure 10

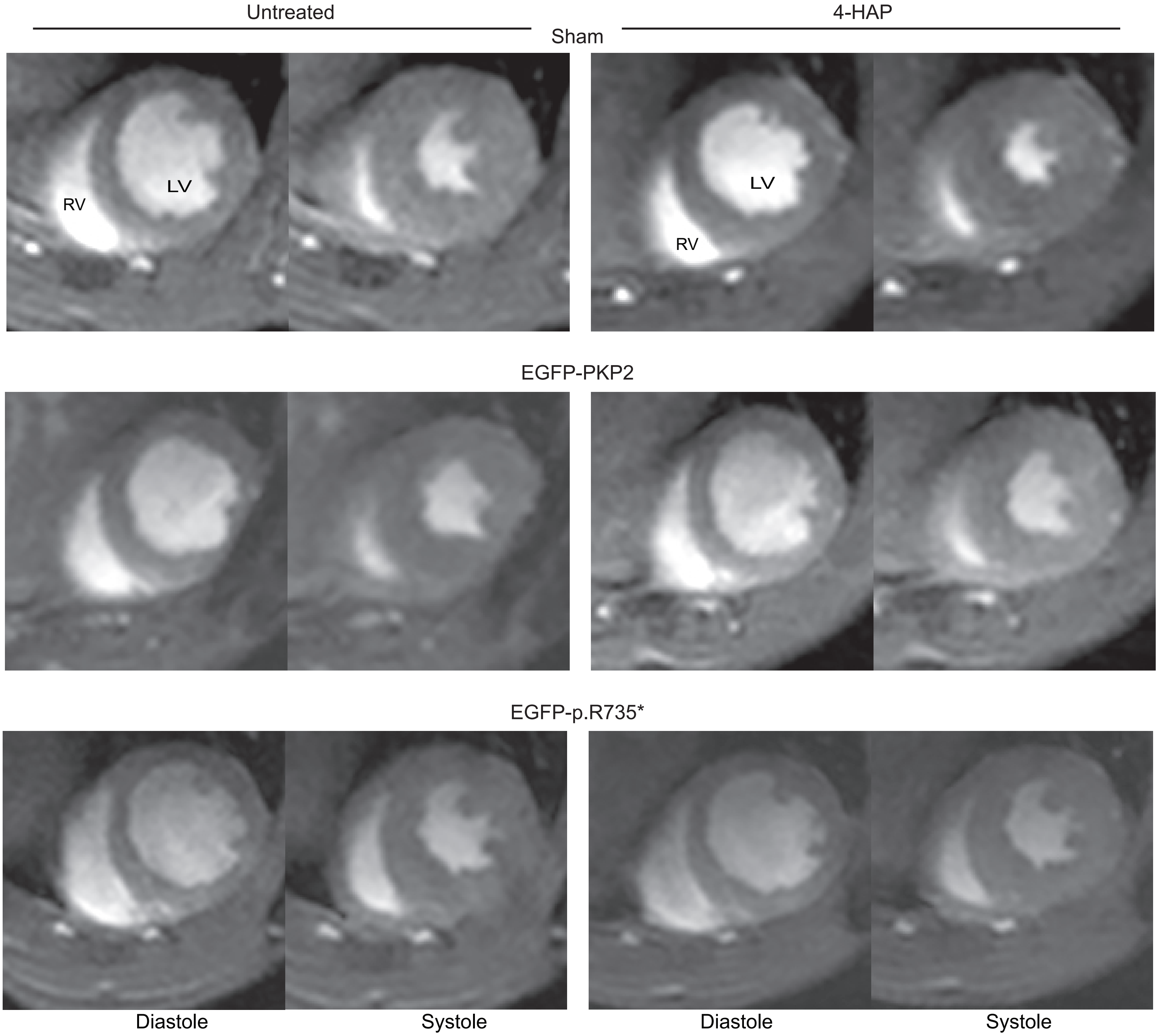

Mean ± SD	RV-EDV [ul]	RV-ESV [µl]	RV-EF [%]	LV-EDV [µl]	LV-ESV [µl]	LV-EF [%]
**Sham**	31.00 ± 2.89	11.17 ± 2.71	64.33 ± 5.68	55.00 ± 5.66	16.00 ± 3.90	70.83 ± 5.27
**EGFP-PKP2**	35.13 ± 6.62	14.38 ± 4.63	59.50 ± 7.17	57.75 ± 10.36	18.13 ± 7.32	69.75 ± 7.72
**EGFP-p.R735***	43.58 ± 8.59	22.25 ± 6.37	49.08 ± 7.53	66.17 ± 9.81	20.00 ± 5.95	70.25 ± 6.34
**Sham / 4-HAP**	28.83 ± 3.66	11.00 ± 1.27	61.83 ± 3.43	57.17 ± 4.54	15.83 ± 3.92	72.83 ± 5.57
**EGFP-PKP2 / 4-HAP**	36.75 ± 5.70	13.38 ± 3.07	63.38 ± 6.59	57.88 ± 11.75	17.75 ± 5.45	69.75 ± 4.27
**EGFP-p.R735* / 4-HAP**	38.67 ± 10.30	15.92 ± 3.26	58.50 ± 4.03	63.83 ± 13.13	19.33 ± 6.73	70.67 ± 8.14

Supplemental Fig. 10 Activation of MYH10 corrects cardiac defects observed in PKP2 mutant cardiomyocytes
Upper panel illustrating representative short-axis cardiac MRI images taken at the end of diastole and systole in hearts of non-transduced (sham) and or AAV-EGFP-PKP2 or AAV-EGFP-PKP2-p.R735* transduced mice, treated or not with 4-HAP for 1 week. Lower panel shows MRI quantifications of EDV: End Diastolic Volume; ESV: End Systolic Volume; RV: Right ventricle; LV: Left ventricle; EF: ejection fraction in anesthetized mice. Data are presented as mean ± sd; n=8 and 12 animals respectively. RV: right ventricle; LV: left ventricle; EDV: end diastolic volume; ESV: end systolic volume; EF: ejection fraction. Source data are provided as a source data file.
