# Supplementary material for: MYH10 activation rescues contractile defects in arrhythmogenic cardiomyopathy (ACM)
Source: Nat Commun. 2023 Oct 13;14:6461. doi: 10.1038/s41467-023-41981-5 (PMC10575922; doi:10.1038/s41467-023-41981-5)
Supplement: Supplementary file 3 — Reporting Summary [file 41467_2023_41981_MOESM3_ESM.pdf]

nature portfolio

Corresponding author(s): Juan A Bernal

Last updated by author(s): Sep 9, 2023

Reporting Summary

Nature Portfolio wishes to improve the reproducibility of the work that we publish. This form provides structure for consistency and transparency in reporting. For further information on Nature Portfolio policies, see our Editorial Policies and the Editorial Policy Checklist.

Statistics

For all statistical analyses, confirm that the following items are present in the figure legend, table legend, main text, or Methods section.

n/a	Confirmed	
☐	☒	The exact sample size (*n*) for each experimental group/condition, given as a discrete number and unit of measurement
☐	☒	A statement on whether measurements were taken from distinct samples or whether the same sample was measured repeatedly
☐	☒	The statistical test(s) used AND whether they are one- or two-sided<br>*Only common tests should be described solely by name; describe more complex techniques in the Methods section.*
☒	☐	A description of all covariates tested
☐	☒	A description of any assumptions or corrections, such as tests of normality and adjustment for multiple comparisons
☐	☒	A full description of the statistical parameters including central tendency (e.g. means) or other basic estimates (e.g. regression coefficient) AND variation (e.g. standard deviation) or associated estimates of uncertainty (e.g. confidence intervals)
☐	☒	For null hypothesis testing, the test statistic (e.g. *F*, *t*, *r*) with confidence intervals, effect sizes, degrees of freedom and *P* value noted<br>*Give P values as exact values whenever suitable.*
☒	☐	For Bayesian analysis, information on the choice of priors and Markov chain Monte Carlo settings
☒	☐	For hierarchical and complex designs, identification of the appropriate level for tests and full reporting of outcomes
☒	☐	Estimates of effect sizes (e.g. Cohen's *d*, Pearson's *r*), indicating how they were calculated

Our web collection on statistics for biologists contains articles on many of the points above.

Software and code

Policy information about availability of computer code

Data collection	All software collection is commercially available and described detailed in the Methods section.
Data analysis	NA

For manuscripts utilizing custom algorithms or software that are central to the research but not yet described in published literature, software must be made available to editors and reviewers. We strongly encourage code deposition in a community repository (e.g. GitHub). See the Nature Portfolio guidelines for submitting code & software for further information.

Data

Policy information about availability of data

All manuscripts must include a data availability statement. This statement should provide the following information, where applicable:

- Accession codes, unique identifiers, or web links for publicly available datasets
- A description of any restrictions on data availability
- For clinical datasets or third party data, please ensure that the statement adheres to our policy

All the data supporting this work are available within the article and its supplementary information files, or can be obtained from the corresponding author upon reasonable request.

Research involving human participants, their data, or biological material

Policy information about studies with human participants or human data. See also policy information about sex, gender (identity/presentation), and sexual orientation and race, ethnicity and racism.

Reporting on sex and gender	N/A
Reporting on race, ethnicity, or other socially relevant groupings	N/A
Population characteristics	N/A
Recruitment	N/A
Ethics oversight	N/A

Note that full information on the approval of the study protocol must also be provided in the manuscript.

Field-specific reporting

Please select the one below that is the best fit for your research. If you are not sure, read the appropriate sections before making your selection.

[x] Life sciences [ ] Behavioural & social sciences [ ] Ecological, evolutionary & environmental sciences

For a reference copy of the document with all sections, see nature.com/documents/nr-reporting-summary-flat.pdf

Life sciences study design

All studies must disclose on these points even when the disclosure is negative.

Sample size	Formal sample size calculation was not performed for mice experiments. Sample size was chosen based on previous studies carried out in the laboratory that showed an "n" where biologically relevant differences between the study groups could be observed. See examples at: (https://doi.org/10.1038/s44161-022-00145-2) or (https://doi.org/10.1016/j.jacc.2015.01.045)<br>For in vitro preliminary studies, the GRANMO sample calculator was used (https://www.imim.es/ofertadeserveis/software-public/granmo/)
Data exclusions	No data were excluded to reflect the biological variability in our AAV-derived mice models, which is also observed in patients suffering from ACM.
Replication	Reproducibility of results were confirmed by repeating the experiments independently by different researchers. All attemps at replication were successful. The in vivo experimental work was verified by using at least n=3 mice for all experiments. For experiments not involving mice at least 3 biological replicates and 2 technical replicates were used with similar results.
Randomization	Sibling male C57BL6 mice were randomly assigned to experimental groups (Research Randomizer web page https://www.randomizer.org) to be transduced with different AAVs.<br>In experiments including neonatal cardiomyocytes segregated by sex, cells were distributed into wells with the same number of cells and the selection of wells to be transfected with AAV was done randomly.
Blinding	In experiment involving mice investigators were blinded at the time of analysis but not at data collection. Data collection could not be performed blinded because it was necessary to be able to differentially identify data form animals infected with different AAVs.<br>Imaging and cellular and biochemical experiments were not performed in a blinding manner because the same investigator was doing group allocation during data collection. Nonetheless, for comparisons between groups, data were extracted automatically from the softwares used, so no subjective judgment was involved at any step. All imaging analysis was performed blindly.

Reporting for specific materials, systems and methods

We require information from authors about some types of materials, experimental systems and methods used in many studies. Here, indicate whether each material, system or method listed is relevant to your study. If you are not sure if a list item applies to your research, read the appropriate section before selecting a response.

Materials & experimental systems

n/a	Involved in the study
☐	☒ Antibodies
☐	☒ Eukaryotic cell lines
☒	☐ Palaeontology and archaeology
☐	☒ Animals and other organisms
☒	☐ Clinical data
☒	☐ Dual use research of concern
☒	☐ Plants

Methods

n/a	Involved in the study
☒	☐ ChIP-seq
☒	☐ Flow cytometry
☒	☐ MRI-based neuroimaging

Antibodies

Antibodies used

- For immunohistochemistry analysis GFP antibody (R1091P, ORIGENE) was used at the dilution 1:500.
- For cell fractionation analysis, PKP2 antibody (Everest Biotech, EB10841), N-cadherin antibody (Santa Cruz, sc-59987; Lot Number: K1017), GAPDH antibody (6C5) (Santa Cruz Biotechnology, sc-32233) were used at the dilution 1:1000.
- For Co-immunoprecipitation analysis Myh10 (Cell Signaling Technology, 3404S; Lot Number: 3) and Living colours A.v. Monoclonal antibody (Takara Bio Clontech, 632381; Lot Number: a8034133) antibodies were used at the dilution 1:1000.
- IgG1 EGFP antibody (Monoclonal antibody CNIO; Lot Number: 193) was used at the dilution 1:100. In all cases, secondary antibodies were HRP anti-mouse (ABIN6699027, Antibodies online) and anti-rabbit (ABIN5563398, Antibodies online), as appropriate.
- For western blot analysis Living Colors® A.v. Monoclonal Antibody (JL-8), dilution 1:1000. (Takara Bio Clontech, 632381; lot number: a8034133), GAPDH antibody (6C5) dilution 1:2000, (Santa Cruz Biotechnology, sc-32233; Lot Number: E2419) and secondary antibody HRP anti-mouse (NA931V, Amersham, 1:10000) were used.

Validation

All antibodies used for this work were purchased from validated commercial sources. Information on each antibody and their validated application is available at the manufacturer´s website. We only used these validated applications.

- GFP antibody (R1091P, ORIGENE) is validated for human, mouse, rat species and for Elisa, IF, IHC, WB applications
-Living Colors® A.v. Monoclonal Antibody (JL-8) is validated for African green monkey, human, mouse, rat, fruit fly, thale cress, rabbit species and for WB, IHC, IC, IP, IHC-P applications.
-GAPDH antibody (6C5) is validated for detection of GAPDH of mouse, rat, human, rabbit and Xenopus species and for Western Blotting , immunoprecipitation and immunofluorescence applications.
- Goat Anti-Plakophilin 2 (EB10841, Everest) is validated for mouse and human species and for Elisa, western blot and IHC applications
- N-cadherin (sc-59987, Santa Cruz) is validated for mouse, rat, human species and for WB, IP, IFand IHC applications.
- GAPDH antibody (sc-32233) is validated for mouse, rat, human, rabbit and Xenopus species for WB, IP and IF applications
- Myosin IIb Antibody (3404S, Cell Signaling Technology) is validated for human, mouse and monkey species for WB, IF-IC applications.
- IgG1 EGFP antibody (Monoclonal antibody National Cancer Research Center (CNIO)) reacts with Jellyfish if WB, IP, IF, Elisa, IHC-P, IHC-F applications

Eukaryotic cell lines

Policy information about cell lines and Sex and Gender in Research

Cell line source(s)

HEK293T (ATCC, CRL-3216), HL-1 (Sigma-Aldrich) cell lines, were obtained through American Type Culture Collection (ATCC) and Sigma-Aldrich company.
Neonatal cardiomyocytes were isolated by the researchers.

Authentication

HL-1 cell line was directly ordered from the supplier and characteristic twitching at high cellular density could be observed. Derived stable cell lines maintained this phenotype at different levels. HEK293T (ATCC, CRL-3216) cells once received from the supplier maintained their ability to produce AAV and were not further authenticated.

Mycoplasma contamination

Cells were routinely tested and confirmed negative for mycoplasma in house by using a Mycoplasma Test from Lonza (MycoAlert PLUS Mycoplasma Detection kit, LT07-318)

Commonly misidentified lines
(See ICLAC register)

No cell lines in this study are listed in the commonly misidentified (ICLAC)

Animals and other research organisms

Policy information about studies involving animals; ARRIVE guidelines recommended for reporting animal research, and Sex and Gender in Research

Laboratory animals

C57BL/6J mice were obtained from Charles Rives Laboratories. Four to six week-old and age-matched male mice were used in all AAV transduction experiments, and were analysed at the indicated times. Neonatal Cardiomyocytes from both sex (C57BL/6J, Charles

	Rives Laboratories) have been used for all mechanistic assay, and no differences have been observed between males and females.
Wild animals	No wild animals were used in this study.
Reporting on sex	ACM affects mainly men. To avoid gender variable, in this project we have analyzed only male mice. Female mice are planning to analyze.
Field-collected samples	This study did not involved samples collection from the field.
Ethics oversight	All animal procedures followed the guidelines from Directive 2010/63/EU of the European Parliament on the protection of animals used for scientific purposes. Animal experiments were carried out in accordance with the CNIC Institutional Ethics Committee recommendations and were approved by the Animal Experimentation Committee (Scientific Procedure) of Comunidad de Madrid (project number PROEX 019/17)

Note that full information on the approval of the study protocol must also be provided in the manuscript.
